# Supplementary material for: Plant guttation provides nutrient-rich food for insects
Source: Proc Biol Sci. 2020 Sep 16;287(1935):20201080. doi: 10.1098/rspb.2020.1080 (PMC7542811; doi:10.1098/rspb.2020.1080)
Supplement: Supplementary figures and tables [file rspb20201080supp1.docx]

**Electronic Supplementary Material**

**Figure S1**. Maximum, mean, and minimum ambient (a) and soil (b) temperatures, relative humidity (c), and solar radiation (d) in highbush blueberry (*Vaccinium corymbosum*) fields from 02 May until 18 July 2019.

**Figure S2**. Correlations between abiotic conditions (ambient (a) and soil (b) temperatures, relative humidity (c), and solar radiation (d)) and the percentage of highbush blueberry (*Vaccinium corymbosum*) leaves with guttation drops throughout the season. Dots are means ± SE. Figures show r and *P* values from the Pearson correlation test.

**Table S1**. Kaplan-Meier survival analyses for the differences in longevity of the insects studied.

**Table S2**. Generalized linear model testing for differences in insect egg load.

**Table S3**. Results from generalized linear mixed models on the percentage of highbush blueberry (*Vaccinium corymbosum*) leaves with guttation and the number of guttation drops per leaf.

**Table S4.** Abiotic conditions (mean ± SE) at different times of day*.*

**Table S5.** Daily and seasonal counts of arthropods visiting highbush blueberry (*Vaccinium corymbosum*) guttation leaf drops throughout the blueberry growing season under field conditions.

**Table S6.** Number of arthropods captured on sticky traps near plants with (guttation) and without (control) guttation drops under field conditions.

**Table S7**. Sugar (g/ml) and protein (mg/ml) concentrations reported in guttation droplets from different crops.


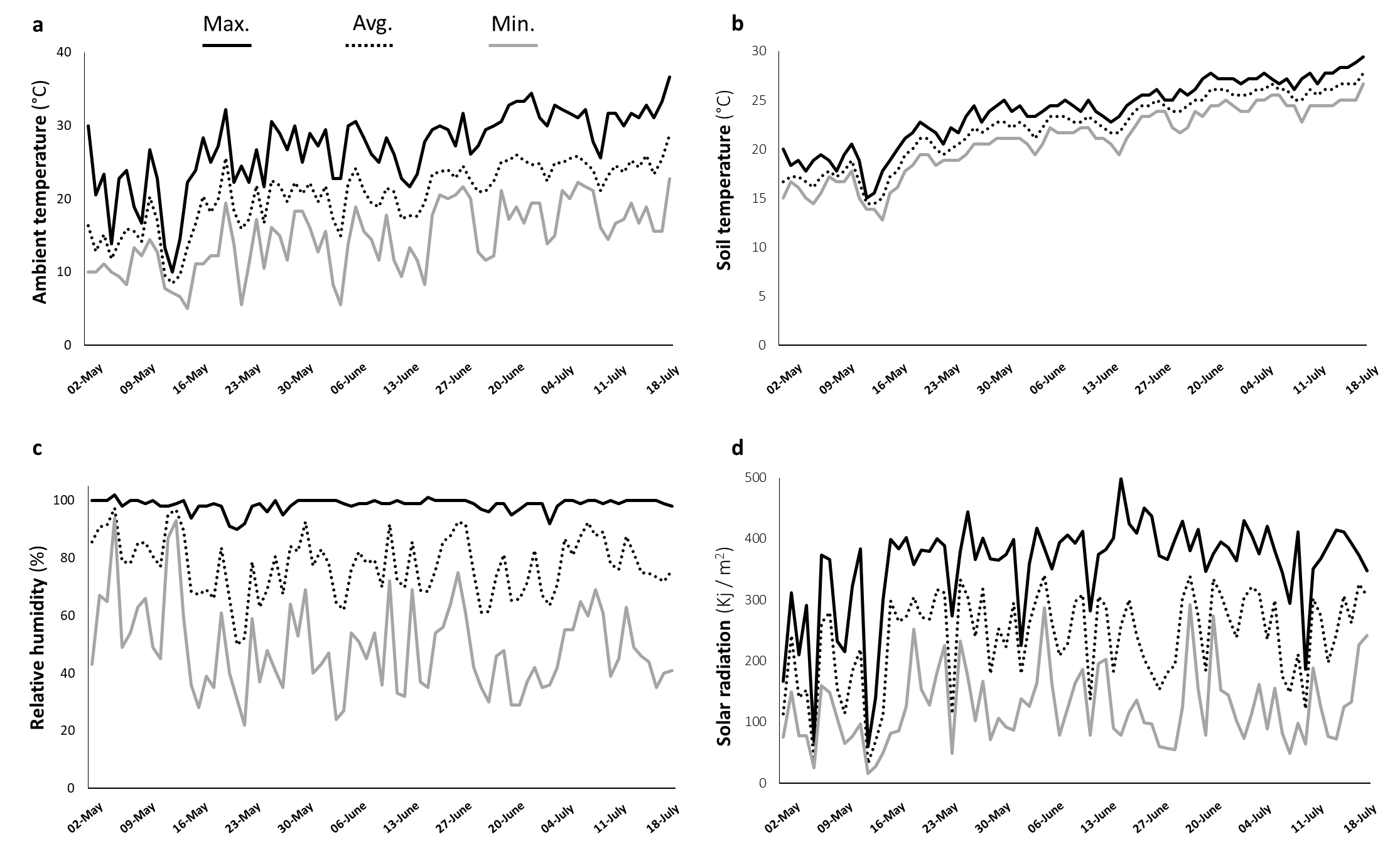


**Figure S1**.


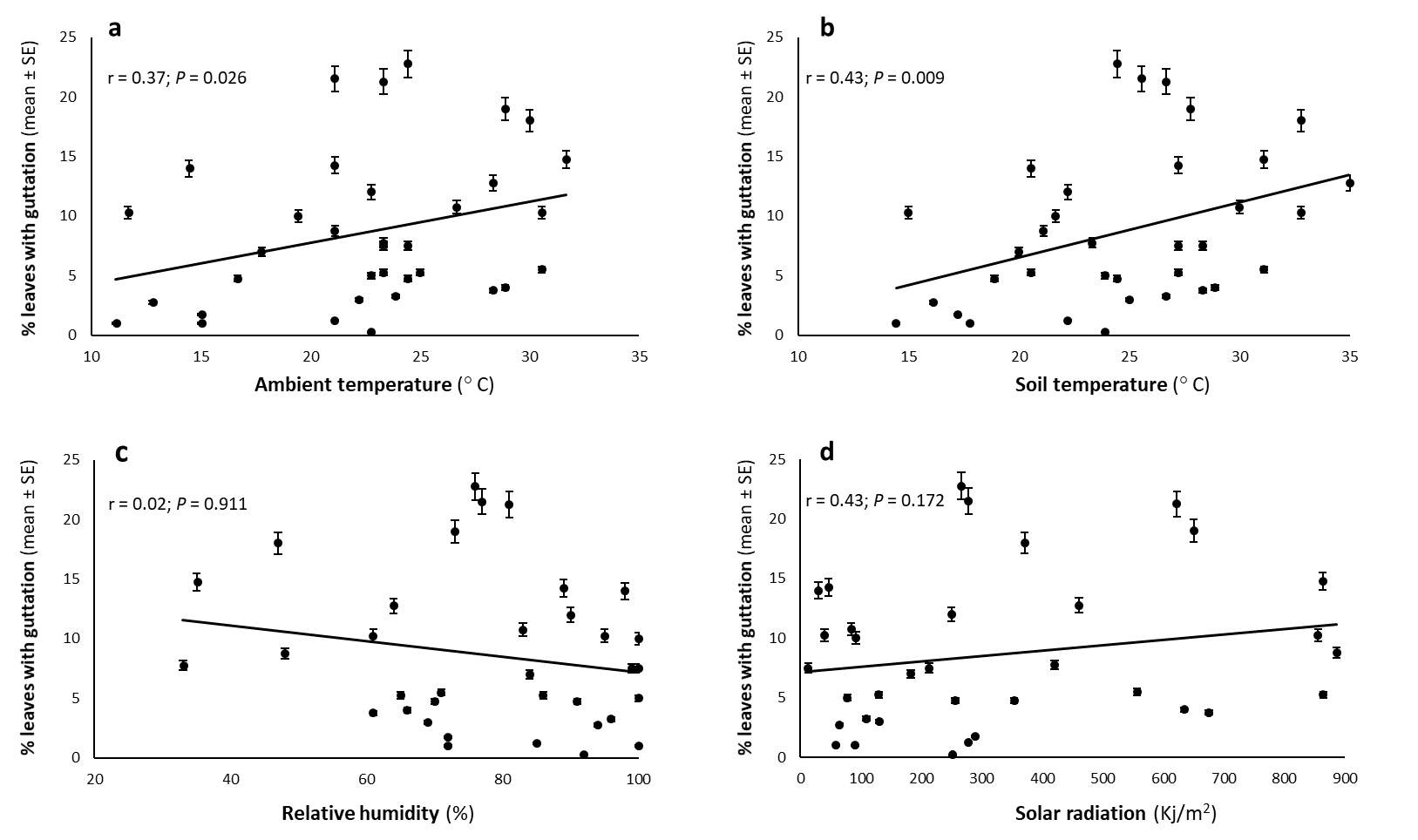


**Figure S2**.

**Table S1**.

| **Insect*^a^*** | **Sex** | Statistics | | |
| --- | --- | --- | --- | --- |
|  |  | *X*^2^ | df | *P* value*^b^* |
| *Drosophila suzukii* | Females | 672.7 | 4 | **<0.001** |
|  | Males | 426.3 | 4 | **<0.001** |
| *Aphidius ervi* | Females | 245.4 | 4 | **<0.001** |
|  | Males | 174.3 | 4 | **<0.001** |
| *Chrysoperla rufilabris* | Females | 762.5 | 4 | **<0.001** |
|  | Males | 594.4 | 4 | **<0.001** |

*^a^*Herbivore *Drosophila suzukii*, the generalist predator *Chrysoperla rufilabris*, and the parasitic wasp *Aphidius ervi* among five diets: guttation, sugar-only, protein-only, sugar plus protein, and water-only. Data were analyzed separately for males and females.

*^b^*Significant *P* values at α = 0.05 are shown in bold.

**Table S2.**

| **Insect*^a^*** | **Day 1** | | | **Day 3** | | | | **Day 7** | | |
| --- | --- | --- | --- | --- | --- | --- | --- | --- | --- | --- |
|  | *F* | df | *P* value | *F* | df | *P* value | *F* | | df | *P* value |
| *Drosophila suzukii* | 73.7 | 4 | **<0.001** | 123.9 | 4 | **<0.001** | 36.56 | | 2 | **<0.001** |
| *Aphidius ervi* | 48.7 | 4 | **0.008** | 344.2 | 4 | **<0.001** | 130.8 | | 2 | **<0.001** |
| *Chrysoperla rufilabris* | 12.8 | 4 | **0.012** | 11.9 | 3 | **0.008** | 11.7 | | 2 | **0.003** |

*^a^*The herbivore *Drosophila suzukii*, the generalist predator *Chrysoperla rufilabris*, and the parasitic wasp *Aphidius ervi* fed on five different diets: guttation, sugar-only, protein-only, sugar plus protein, and water-only. Data were analyzed separately for egg loads 1, 3, and 7 days after adult emergence. Significant *P* values at α = 0.05 are shown in bold.

**Table S3**.

| **Effect** | **Statistics by measurement** | | | | | |
| --- | --- | --- | --- | --- | --- | --- |
|  | **Percentage of leaves with guttation** | | | **Drops per leaf with guttation** | | |
|  | *F* | df | *P* value | *F* | df | *P* value |
| **Model** | 24.9 | 35 | **<0.001** | 26.1 | 35 | **<0.001** |
| **Date** | 50.2 | 11 | **<0.001** | 34.9 | 11 | **<0.001** |
| **Time** | 18.8 | 2 | **<0.001** | 7.2 | 2 | **0.001** |
| **Date*Time** | 10.8 | 22 | **<0.001** | 10.9 | 22 | **<0.001** |

*^a^*The models included the effects of ‘date’ (sampling date), and ‘time’ (8 am, 1 pm, and 6 pm), and the interaction as fixed factors; ‘field’ as a covariate; and ‘bush’ (replicate) as a random factor. Significant *P* values at α = 0.05 are shown in bold.

**Table S4**.

| **Abiotic factor** | **Values by time of day** | | | **Statistics** | | |
| --- | --- | --- | --- | --- | --- | --- |
|  | **8 am** | **1 pm** | **6 pm** | *F* | df | *P* value |
| Ambient temperature (°C) | 18.8 ± 1.6 b | 24.8 ± 1.6 a | 23.6 ± 1.1 a | 10.8 | 2 | **0.004** |
| Soil temperature (°C) | 20.9 ± 1.2 b | 25.7 ± 1.5 a | 26.9 ± 1.3 a | 12.4 | 2 | **0.002** |
| Relative humidity (%) | 92 ± 2.6 a | 66 ± 3.9 b | 75.2 ± 5.8 b | 20.3 | 2 | **< 0.001** |
| Solar radiation (Kj/m^2^) | 132.1 ± 27.1 c | 667.9 ± 68.9 a | 231.3 ± 43.6 b | 35.6 | 2 | **< 0.001** |

*^a^*Different letters indicate differences among times of day for a particular abiotic condition (generalized linear models). Significant *P* values at α = 0.05 are shown in bold.

**Table S5**.

| **Arthropod fauna** | **Family or group** | **Counts by month and time** | | | | | | | | | **Seasonal count by time** | | | | **Total counts** | |
| --- | --- | --- | --- | --- | --- | --- | --- | --- | --- | --- | --- | --- | --- | --- | --- | --- |
|  |  | **May** | | | **June** | | | **July** | | |  |  |  |  |  |  |
|  |  | 8 am | 1 pm | 6 pm | 8 am | 1 pm | 6 pm | 8 am | 1 pm | 6 pm | 8 am | 1 pm | 6 pm |  | |  |
| **Beneficial taxa** | Anthocoridae | 0 | 0 | 0 | 4 | 6 | 5 | 0 | 0 | 2 | 4 | 6 | 7 | 17 | |  |
|  | Apidae | 0 | 0 | 0 | 10 | 14 | 11 | 7 | 1 | 5 | 17 | 15 | 16 | 48 | |  |
|  | Cecidomyiidae | 0 | 0 | 0 | 3 | 2 | 6 | 9 | 5 | 10 | 12 | 7 | 16 | 35 | |  |
|  | Chrysopidae | 12 | 14 | 10 | 44 | 23 | 30 | 4 | 13 | 8 | 60 | 50 | 48 | 158 | |  |
|  | Coccinellidae | 0 | 0 | 0 | 1 | 0 | 1 | 1 | 0 | 0 | 2 | 0 | 1 | 3 | |  |
|  | Dolichopodidae | 0 | 1 | 3 | 9 | 17 | 12 | 11 | 12 | 5 | 20 | 30 | 20 | 70 | |  |
|  | Syrphidae | 5 | 11 | 2 | 13 | 7 | 6 | 3 | 2 | 6 | 21 | 20 | 14 | 55 | |  |
|  | Formicidae | 23 | 31 | 29 | 26 | 32 | 36 | 5 | 6 | 11 | 54 | 69 | 76 | 199 | |  |
|  | Thomisidae | 7 | 20 | 14 | 20 | 31 | 25 | 5 | 13 | 9 | 32 | 64 | 48 | 144 | |  |
|  | Staphylinidae | 1 | 0 | 0 | 0 | 1 | 1 | 0 | 0 | 0 | 1 | 1 | 1 | 3 | |  |
|  | Parasitic wasps | 5 | 18 | 7 | 13 | 12 | 7 | 3 | 4 | 6 | 21 | 34 | 20 | 75 | |  |
| **Herbivores** | Drosophilidae | 0 | 4 | 4 | 20 | 20 | 20 | 30 | 21 | 65 | 50 | 45 | 89 | 184 | |  |
|  | Thysanoptera | 0 | 1 | 0 | 20 | 20 | 22 | 0 | 0 | 0 | 20 | 21 | 22 | 63 | |  |
|  | Curculionidae | 0 | 0 | 0 | 0 | 0 | 0 | 0 | 0 | 3 | 0 | 0 | 3 | 3 | |  |
| **Total** | | 53 | 100 | 69 | 183 | 185 | 182 | 78 | 77 | 130 | 314 | 362 | 381 | 1,057 | |  |

**Table S6.**

| **Arthropod fauna** | **Family or group** | **No. of arthropods by treatment** | | ***t*-tests*^a^***  (*t*; df; *P* value) |
| --- | --- | --- | --- | --- |
|  |  | **Control** | **Guttation** |  |
| **Beneficial taxa** | Anthocoridae | 1 | 7 | - |
|  | Cecidomyiidae | 93 | 186 | 3.15; 78; **0.001** |
|  | Coccinellidae | 2 | 4 | - |
|  | Dolichopodidae | 109 | 158 | 2.21; 78; **0.030** |
|  | Formicidae | 9 | 4 | - |
|  | Miridae | 4 | 11 | - |
|  | Parasitic wasps | 99 | 177 | 4.01; 78; **<0.001** |
|  | Syrphidae | 1 | 3 | - |
|  | Staphylinidae | 9 | 18 | - |
|  | Thomisidae | 7 | 15 | - |
|  | Carabidae | 0 | 2 | - |
|  | **Total** | 334 | 585 | 4.89; 78; **<0.001** |
| **Herbivores** | Agromizidae | 3 | 6 | - |
|  | Aphididae | 53 | 29 | 2.62; 78; **0.011** |
|  | Cicadellidae | 55 | 37 | 1.24; 78; 0.217 |
|  | Curculionidae | 1 | 0 |  |
|  | Delphacidae | 0 | 1 | - |
|  | Derbidae | 0 | 1 | - |
|  | Diaspididae | 26 | 35 | 0.68; 78; 0.499 |
|  | Drosophilidae | 523 | 539 | 0.51; 78; 0.881 |
|  | Fulgoridae | 0 | 1 | - |
|  | Gryllidae | 3 | 2 | - |
|  | Nitidulidae | 5 | 1 | - |
|  | Thysanoptera | 109 | 115 | 0.23; 78; 0.822 |
|  | Tephritidae | 2 | 0 | - |
|  | **Total** | 780 | 767 | 0.89; 78; 0.930 |
| **Others** | Culicidae | 73 | 31 | 2.29; 78; **0.024** |
|  | Tabanidae | 2 | 2 | - |
|  | **Total** | 75 | 33 | 2.02; 78; **0.037** |

*^a^*Only the most abundant arthropod taxa were analyzed statistically. Significant *P* values at α = 0.05 are shown in bold.

**Table S7.**

| **Crop** | **Sugars**  **(g/ml)** | **Protein**  **(mg/ml)** | **Reference** |
| --- | --- | --- | --- |
| Rye | 0.0000484 | 0.0062 | [24] |
| Wheat | 0.0000271 | 0.0027 |  |
| Barley | 0.0000498 | 0.0143 |  |
| Cucumber | - | 0.15 | [30] |
| Strawberry | - | 0.1 |  |
| Tomato | - | 0.1 |  |
| Tobacco | - | 30 | [28] |
| Barley | - | 5 | [39] |
| Maize | 0.75 | - | [27] |
| Highbush blueberry | 1.5 | 4.3 | Present study |

(-) Not reported.
